# The Disinfection of Sleeping Cars

**Published:** 1902-10

**Authors:** 


### The Disinfection of Sleeping Cars.

Through carelessness or inability to prevent it, tuberculous passengers foul the blankets on their beds and the curtains of their berths with sputum. R. J. Wilson, instructor in bacteriology, New York University, has tried some experiments in sleeping car disinfectants with a view, first, to get good disinfection. and, second, to do it so that the disinfected car is made fit for service in the shortest possible time. After describing trials of different quantities of 40 per cent. formalin and formaldehyde gas, generated directly from methyl-alcohol by passing it over red-hot platinum, he says: "In the fifth experiment formaldehyde gas was generated from methyl-alcohol direct, six litres of alcohol were used and the time of exposure was five hours. There was superficial disinfection without penetration. There was a strong odor of methyl-alcohol in the car for a few hours after disinfection, but not enough to keep it out of service." In reference to the comparative merits of the different forms of disinfection by formaldehyde he says: "The persistence of the formalin odor, after disinfection with formaldehyde gas generated from forty per cent. formalin or paraform, renders these undesirable for car disinfection, while the rapid dissipation of the odor after the use of methyl-alcohol particularly, recommends it to favor for surface disinfection." As to the penetrating power of any form of formaldehyde disinfection his conclusion is: "Penetration can not be counted on, so that all portable articles in the car, especially the blankets, should be disinfected in a chamber where complete penetration is assured."—Editorial note in *Canadian Journal of Medicine and Surgery.*

